# The Contribution of Non-catalytic Carbohydrate Binding Modules to the Activity of Lytic Polysaccharide Monooxygenases*[Fn FN2]

**DOI:** 10.1074/jbc.M115.702365

**Published:** 2016-01-22

**Authors:** Lucy I. Crouch, Aurore Labourel, Paul H. Walton, Gideon J. Davies, Harry J. Gilbert

**Affiliations:** From the ‡ Institute for Cell and Molecular Biosciences, The Medical School Newcastle University, Newcastle upon Tyne NE2 4HH and; the § York Structural Biology Laboratory, Department of Chemistry, University of York, York YO10 5DD, United Kingdom

**Keywords:** carbohydrate-binding protein, enzyme, glycoside hydrolase, plant cell wall, protein engineering, carbohydrate binding modules, lytic polysaccharide monooxygenases, cellulases, lignocellulose degradation

## Abstract

Lignocellulosic biomass is a sustainable industrial substrate. Copper-dependent lytic polysaccharide monooxygenases (LPMOs) contribute to the degradation of lignocellulose and increase the efficiency of biofuel production. LPMOs can contain non-catalytic carbohydrate binding modules (CBMs), but their role in the activity of these enzymes is poorly understood. Here we explored the importance of CBMs in LPMO function. The family 2a CBMs of two monooxygenases, *Cf*LPMO10 and *Tb*LPMO10 from *Cellulomonas fimi* and *Thermobispora bispora*, respectively, were deleted and/or replaced with CBMs from other proteins. The data showed that the CBMs could potentiate and, surprisingly, inhibit LPMO activity, and that these effects were both enzyme-specific and substrate-specific. Removing the natural CBM or introducing *Ct*CBM3a, from the *Clostridium thermocellum* cellulosome scaffoldin CipA, almost abolished the catalytic activity of the LPMOs against the cellulosic substrates. The deleterious effect of CBM removal likely reflects the importance of prolonged presentation of the enzyme on the surface of the substrate for efficient catalytic activity, as only LPMOs appended to CBMs bound tightly to cellulose. The negative impact of *Ct*CBM3a is in sharp contrast with the capacity of this binding module to potentiate the activity of a range of glycoside hydrolases including cellulases. The deletion of the endogenous CBM from *Cf*LPMO10 or the introduction of a family 10 CBM from *Cellvibrio japonicus* LPMO10B into *Tb*LPMO10 influenced the quantity of non-oxidized products generated, demonstrating that CBMs can modulate the mode of action of LPMOs. This study demonstrates that engineered LPMO-CBM hybrids can display enhanced industrially relevant oxygenations.

## Introduction

Plant biomass represents an important biological and industrial substrate. These highly crystalline composite structures are degraded and utilized by microorganisms that occupy important ecological niches, while the process makes an important contribution to the carbon cycle (1). Lignocellulosic degradation is also of continued interest to environmentally sensitive industries such as the biofuels and biorefinery sectors, where the use of environmentally sustainable substrates is of increasing importance. Given that lignocellulose represents the most abundant source of organic carbon in the biosphere, these composite substrates have substantial industrial potential (2).

Cellulose, a polymer of β-1,4-linked glucose, is the most abundant component of plant biomass. The polysaccharide is degraded into its monosaccharide by the synergistic action of *exo*/processive-acting cellobiohydrolases and *endo*-β-1,4-glucanses, whereas β-glucosidases reduce product inhibition and complete the saccharification process (see Ref. 1) for review). Cellulose is a crystalline molecule that is highly recalcitrant to biological degradation. It was recognized at the inception of cellulase research as early as 1950 that a factor, termed C_1_, was required to make cellulose accessible to the hydrolytic cellulases (3). In 2010, a solubilizing factor, consistent with the C1 hypothesis, was unveiled, and these enzymes are now known as the lytic polysaccharide monooxygenases (LPMOs).^3^ They were demonstrated to have oxidase activity and in 2011 were shown to be copper-dependent monooxygenases (4). These enzymes have been shown to contribute significantly to biomass degradation (5). LPMOs were initially identified in chitin- and cellulose-degrading systems of aerobic microorganisms (6, 7). More recently, LPMOs have also been identified that attack starch (8), oligosaccharides, and soluble glycans such as xyloglucan (9–11). LPMOs are currently grouped into sequence-based “auxiliary activity” families AA9, AA10, AA11, and AA13 on the CAZY database (12), with AA9 and AA10 containing cellulose specific fungal and bacterial enzymes, respectively. LPMOs have been described that exclusively oxidize C1, or C4, or both C1 and C4 (10, 13).

Enzymes that attack cellulose and, more generally, plant cell walls, frequently contain non-catalytic carbohydrate binding modules or CBMs (see Refs. 14 and 15 for review). CBMs have also been grouped into sequence-based families on the CAZy database and, based on ligand specificity, into three types (14, 15) dependent on whether they bind to crystalline ligands (type A), the internal regions of glycan chains (type B), or the termini of polysaccharides and oligosaccharides (type C). These modules, which were first identified in cellulases (16, 17), recruit their cognate enzymes into close proximity with their target substrates and thus promote catalysis (18, 19). It has also been proposed that CBMs can direct enzymes to regions of the plant cell wall that are particularly accessible to biological attack (20, 21), whereas it has also been shown that these modules can also modulate enzyme specificity (22).

The roles of CBMs in the function of glycoside hydrolases have been widely explored. These modules, however, are appended to other enzymes that attack recalcitrant substrates. For example, CBMs are present in ∼30% of LPMOs, and are located in families consistent with the specificity of the cognate enzymes (23). Thus, LPMOs that target cellulose contain type A CBMs from families 1 (fungal enzymes) or 2a or 10 (on bacterial enzymes) that bind to crystalline forms of the polysaccharide. Chitin-specific LPMOs also contain type A CBMs, principally from families 5 and 12, but also 2a, which target the GlcNAc-based glycan. The AA13 LPMOs that cleave starch contain family 20 CBMs that are known to bind to this storage polymer. It is evident that there are significant differences in the topography of the ligands and substrates recognized by type A CBMs and glycoside hydrolases that target cellulose. By contrast, three-dimensional structural data of crystalline cellulose-specific LPMOs indicate that both the ligand binding site of the type A CBMs (24–27) and the substrate binding site of the catalytic domains display a planar surface (4, 13, 28), although no ligand complexes are yet available. This conservation in cellulose recognition may point to substantial synergy between the catalytic and non-catalytic modules in LPMOs. Indeed, the CBMs may play a more direct role in presenting substrate to the active site of cellulose-specific LPMOs than occurs in multi-modular cellulases.

Currently, there is a paucity of information on the role of CBMs in the activity of LPMOs. Removal of the CBM from two AA10 enzymes caused a modest ∼2-fold reduction in activity against phosphoric acid-swollen cellulose (PASC) (13) and Avicel (29), respectively. The influence of a CBM1 on the activity of a *Neurospora* AA9 was assessed against a range of substrates. CBM deletion did not affect enzyme activity against PASC but resulted in a 2-fold reduction in catalytic rate against xyloglucan (11). The modest effect of the CBMs is surprising given the likely cooperativity in substrate binding displayed by the catalytic and non-catalytic modules. Indeed, the potentiation of LPMO activity by CBMs is significantly less than observed in plant cell wall-degrading glycoside hydrolases and polysaccharide lyases including cellulases (18, 19, 30, 31). It is evident that a more detailed analysis of the role of CBMs in LPMO action is required.

In view of the paucity of data described above, the aim of this study was to evaluate the capacity of diverse CBMs to modulate the catalytic activity of cellulose-specific LPMOs. The data presented here showed that the influence of CBMs was both enzyme-specific and substrate-specific and, in the case of the LPMO that oxidized C4 and C1, appeared to modulate the mode of action of the oxygenase. CBM fusions showed that these modules played a more precise role in enzyme function than observed in cellulases and that the “wrong” CBM/LPMO pair can even be deleterious, with implications for “designer” hybrid enzymes. Cellulose binding studies indicated that prolonged retention of LPMOs on substrate was mediated by their CBMs. This study indicates that the role of CBMs in LPMOs and cellulases is not conserved, and provides insight into how CBM engineering could be deployed to improve the catalytic function of these industrially relevant oxygenases.

## Experimental Procedures

### 

#### 

##### Cloning, Expression, and Purification

The genes encoding AA10s from *Thermobispora bispora* and *Cellulomonas fimi* were synthesized using the Life Technologies GeneArt® service and codon-optimized for *Escherichia coli*. These genes were cloned into pRSETB vector behind the native signal sequence from the *Serratia marcescens* BJL200 CBP21 (32). A silent point mutation was made at the end of the signal sequence to produce an NcoI restriction site to allow easy cloning while maintaining the position of the critical first histidine (6) directly after the signal sequence cleavage site. The full-length proteins are named *Cf*LPMO10 and *Tb*LPMO10. The other constructs were produced by sewing PCR. The AA10-only constructs are dubbed *Cf*LPMO10_CD_ and *Tb*LPMO10_CD_, and the CBM swapping constructs are called *Cf*LPMO10_CD_-*Tb*CBM2a and *Tb*LPMO10_CD_-*Cf*CBM2a. Two CBMs were also appended to each of the AA10s: CBM10 from *Cellvibrio japonicus Cj*LPMO10B and CBM3a from *Clostridium thermocellum* cellulosome-integrating CipA. Expression of the different LPMO constructs was optimized using different *E. coli* strains, and all proteins were expressed as described previously (34). *Cf*LPMO10, *Tb*LPMO10, and *Cf*LPMO10-*Tb*CBM2a were expressed in BL21 (DE3). *Cj*Cel6A and *Cj*Cel5B were produced as described previously (34). *Tb*LPMO10_CD_, *Cf*LPMO10_CD_-CBM10, *Tb*LPMO10_CD_-CBM10, and *Cf*LPMO10_CD_-CBM3a were expressed in BL21 (DE3) pLysS. *Cf*LPMO10_CD_, *Tb*LPMO10_CD_-*Cf*CBM2a, *Tb*LPMO10_CD_-*Ct*CBM3a, *Cj*Cel6A, and *Cj*Cel5B were expressed in Shuffle (35). Cells were harvested and protein was purified as described previously (34).

The gene fragments *Ctcbm3a* and *Cjcbm10* were derived from *C. thermocellum* cellulosome-integrating protein CipA (20) and from *C. japonicus Cj*LPMO10B (38), respectively. *Cfcbm2a* and *Tbcbm2a* were amplified from the plasmids containing the full-length constructs of *Cflpmo10* and *Tblpmo10*, respectively. *Ctcbm3a* and *Tbcmb2a* were cloned into pET21a, and *Cjcbm10* was cloned into pET28b using the restriction site pairs NheI/XhoI. *Cfcbm2a* was cloned into pGEX6P-1 using the restriction site pairs EcoRI/XhoI. The encoded proteins *Tb*CBM2a and *Ct*CBM3a display a C-terminal His_6_ tag, whereas *Cf*CBM2a displays an N-terminal GST tag and a C-terminal His_6_ tag, and *Cj*CBM10 displays a His_6_ tag at both the N terminus and the C terminus. Production of *Tb*CBM2a and *Ct*CBM3a was carried out in *E. coli* strain BL21(DE3), and production of *Cf*CBM2a and *Cj*CBM10 as carried out in Shuffle(DE3). Strains harboring the *Tbcbm2a* and *Ctcbm3a* genes were grown at 37 °C to mid-exponential phase and cooled to 16 °C, and recombinant gene expression was induced using 1 mm isopropyl β-d-thiogalactopyranoside and incubation for a further 15 h. To produce *Cf*CBM2a and *Cj*CBM10, *E. coli* was cultured to mid-exponential phase at 30 °C and cooled to 16 °C, followed by the addition of 0.4 mm isopropyl β-d-thiogalactopyranoside and incubation for a further 15 h. Cell pellets were sonicated, and cell debris was removed by centrifugation. The recombinant proteins were purified as described previously (34). In the particular case of *Cf*CBM2a, the GST tag was cleaved before binding isotherm measurements using the PreScission Protease (GE Healthcare Life Sciences) in 50 mm Tris-HCl, 150 mm NaCl, 1 mm EDTA, 1 mm DTT, pH 7.0 buffer. For all recombinant proteins, a subsequent step of purification was performed by size exclusion chromatography using 20 mm Tris-HCl, 300 mm NaCl, pH 8.0 buffer.

##### LPMO Assays

Time course assays were typically carried out using 1 μm of protein, 0.3 mg/ml of substrate, 1 mm ascorbate, 50 mm phosphate buffer (pH 6), and 0.5 μm copper sulfate. All assays were carried out at 37 °C and 150 rpm. The assays were typically 2 ml, and 200-μl aliquots were taken for every time point. The reaction was stopped by boiling, and the insoluble material was centrifuged out. The soluble part of the assay was either applied directly to high performance anion exchange chromatography (HPAEC) or pretreated with a β-glucosidase before applying to the HPAEC to assess the concentration of gluconic acid.

##### HPAEC

Enzyme reaction products were analyzed using a CARBOPAC^TM^ PA-1 anion exchange column (Dionex) with a CARBOPAC^TM^ PA-1 guard column and run at a rate of 1 ml/min. The loaded samples were usually 40 μl of reaction mixed with 160 μl of filtered water. The exception to this was the synergy samples, which were 5 μl of sample mixed with 195 μl of filtered water. The column was equilibrated with 100 mm NaOH. The reaction products from the LPMO reactions were eluted with a 0–300 mm sodium acetate gradient in 100 mm NaOH. The column was cleaned with 1 m sodium acetate in 100 mm NaOH and then with 500 mm NaOH. For measuring glucuronic acid, the chromatography was performed in 66.7 mm NaOH, and elution of oligosaccharides was achieved using a 0–200 mm sodium acetate gradient in 66.7 mm NaOH.

##### MALDI-TOF Mass Spectrometry

The LPMO assay solutions were spotted at a ratio of 1:1 with a saturated solution of 2,5-dihydroxybenzoic acid onto a polished steel plate. Spots were analyzed on a Bruker ultraflex II MALDI-TOF mass spectrometer in positive reflectron mode. Spectra were visualized and data were analyzed using Bruker flexAnalysis 3.0 and mMass 4.0 (36).

##### Qualitative Cellulose Binding Assays

Proteins (80 μg) were mixed with the following insoluble polysaccharides: 5% (w/v) Avicel, 1% (w/v) PASC, or 0.35% BMCC in a final volume of 200 μl containing 20 mm sodium phosphate buffer, pH 6.0. 10 mm EDTA was added to the samples containing *Cf*LPMO10_CD_ and *Tb*LPMO10_CD_ to avoid any catalytic activity. Tubes were incubated on ice for 1 h with gentle mixing before being centrifuged at 13,000 × *g* for 2 min, and the supernatants (containing the unbound proteins) were carefully removed. The polysaccharide pellets were washed by resuspending in buffer and centrifuged. This step was done twice. Only the supernatant corresponding to the second wash was analyzed on gel. The remaining pellet was finally resuspended in SDS-loading buffer without dye (with a volume equivalent to the unbound fraction) and boiled for 10 min to dissociate any bound protein. 10 μl of unbound, wash, and bound fractions were analyzed by SDS-PAGE on a 12% acrylamide gel.

##### Cellulose Binding Isotherms

The experiments were carried out on ice in 50 mm sodium phosphate buffer, pH 6.0. A range of protein concentrations (0.5–50 μm) was added to the insoluble ligand under test (0.5 mg of Avicel, 0.25 mg of PASC, or 0.2 mg of BMCC), to a final aqueous volume of 500 μl, and the cellulose was kept in suspension by regularly tapping the tubes throughout the assay period (∼1 h). Tubes were then centrifuged at 13,000 × *g* for 10 min, and the supernatant was transferred to a fresh tube, before being centrifuged again to remove any remaining particulates. The *A*_280 nm_ was measured to determine the free protein concentration. Non-linear regression of the isotherm data was obtained using the GraphPad Prism^TM^ software. The one-site model was chosen for our analysis. Each isotherm was repeated at least three times (biological replicates).

##### The Use of Amplex Red to Assess the Function of the LPMO Active Site

Measurements were carried out in a Cary Eclipse Varian Fluorescence Spectrophotometer using 100 mm phosphate buffer (pH 6.0), 50 μm Amplex Red, 30 μm sodium ascorbate, and 7.14 units/ml of horseradish peroxidase in a total volume of 500 μl (37). The LPMO was added at a final concentration of 10 μm, and changes in fluorescence were recorded over 10–15 min using excitation and emission wavelengths of 560 and 585 nm, respectively.

## Results

### 

#### 

##### Characterization of Model LPMOs

**FIGURE 1.**
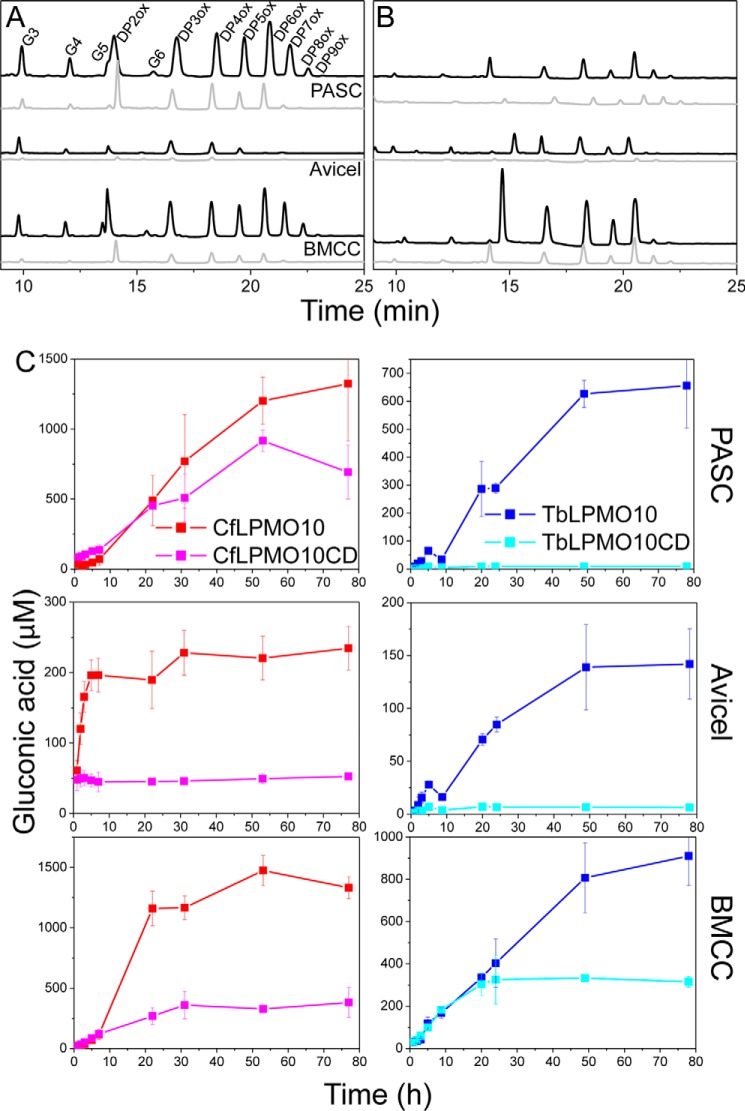
**HPAEC analysis of LPMOs.** *A* and *B*, the HPAEC profiles of the reaction products for *Cf*LPMO10 and *Cf*LPMO10_CD_ (*black* and *gray*, respectively) (*A*) and for *Tb*LPMO10 and *Tb*LPMO10_CD_ (*black* and *gray*, respectively) (*B*). *G3*, *G4*, *G5*, and *G6* are cellotriose, cellotetraose, cellopentaose, and cellohexaose, respectively. *C*, the gluconic acid produced by the full-length constructs and enzymes without CBMs on three different substrates. Oxidized (*DPXox*) and non-oxidized products (*GX*), where *X* is the degree of polymerization (DP), are indicated. *Error bars* indicate means ± S.E.

**FIGURE 2.**
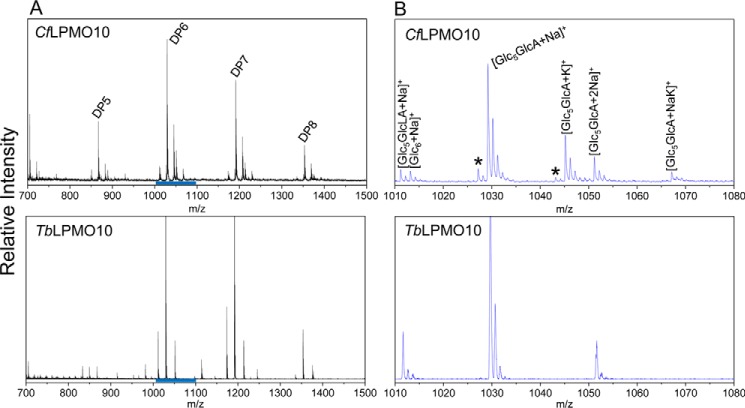
**MALDI-TOF analysis of products released by *Ct*LPMO10 and *Tb*LPMO10.** *A*, the different oligosaccharides generated with DPs indicated. *B*, details of the cellohexaose species. *GlcLA* and *GlcA* represent the lactone and aldonic acid adducts, respectively. The *blue bars* in *A* correspond to the regions detailed in *B*. The *asterisk* indicates the peaks that are indicative of double C1+C4 oxidation, as they are 2 Da smaller than the corresponding C1-oxidized products.

**FIGURE 3.**
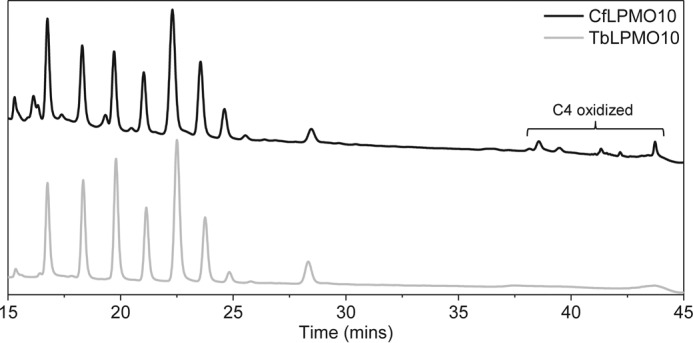
**HPAEC analysis of products released by *Cf*LPMO10 and *Tb*LPMO10 to show evidence for C4 oxidation by the *C. fimi* enzyme.**

A significant cohort of AA10 LPMOs is appended to CBMs (38). To explore the role of CBMs in the catalytic function of AA10 enzymes, the effect of these modules on the activity of two LPMOs, *Tb*LPMO10 and *Cf*LPMO10, was explored. The HPAEC data presented in Fig. 1, *A* and *B*, show that the two enzymes were active against highly crystalline (Avicel and BMCC) and disordered (PASC) forms of cellulose. Both enzymes generated significantly more oligosaccharides from BMCC and PASC than Avicel. This may reflect differences in the surface area of the substrates. BMCC microfibrils are extremely thin when compared with those in Avicel (39), whereas the acid treatment used to generate PASC disrupts the crystalline structure of the polysaccharide, which increases the number of solvent-exposed cellulose chains (40). The two LPMO10s were not active against cello-oligosaccharides and displayed very limited activity against chitins (data not shown). HPAEC and mass spectrometry showed that both enzymes generated a range of oxidized oligosaccharides with a degree of polymerization (DP) ranging primarily from 2 to 7 (Figs. 1, *A* and *B*, and 2). Significantly, *Cf*LPMO10 also generated a series of non-oxidized cello-oligosaccharides with a DP of 2–5. The *C. fimi* enzyme also generated products with a mass 2 Da smaller than both non-oxidized and C1-oxidized cello-oligosaccharides (Fig. 2), indicating that these molecules are C4- and C1+C4-oxidized oligosaccharides, respectively. Furthermore, HPAEC revealed products that eluted in the region associated with C4-oxidized cello-oligosaccharides, first identified by Forsberg *et al.* (13) (Fig. 3).

To determine the rate of enzyme activity against BMCC, PASC, and Avicel, the oxidized reducing end sugar generated by the LPMOs was released from the soluble cello-oligosaccharides by treatment with a nonspecific β-glucosidase. The gluconic acid generated was then quantified by HPAEC. The data, presented in Fig. 1*C* and Table 1, showed that the initial rates of the two enzymes against the three forms of cellulose were very low when compared with cellulases, particularly with PASC as the substrate (34). *Tb*LPMO10 was less active than *Cf*LPMO10 in terms of both the initial rate and the final concentration of gluconic acid produced. This may reflect the thermostable enzyme operating in non-optimal conditions.

**TABLE 1:** **Activity of LPMOs against cellulosic substrates** Activity is presented as initial rates of gluconic acid production (μm gluconic acid/μm protein/h). Values are presented ± standard deviation (to two decimal places).

	PASC	Avicel	BMCC
CfLPMO10	29.1 ± 10.4	21.2 ± 2.38	46.7 ± 6.86
TbLPMO10	14.0 ± 2.79	6.24 ± 1.27	19.4 ± 2.25
CfLPMO10CD	16.4 ± 2.16	ND*^a^*	11.6 ± 3.60
TbLPMO10CD	1.86 ± 0.08	1.27 ± 0.12	20.2 ± 0.25
CfLPMO10-TbCBM	51.2 ± 8.44	30.9 ± 5.99	23.9 ± 3.20
TbLPMO10-CfCBM	2.27 ± 0.78	2.33 ± 0.48	17.1 ± 10.29
CfLPMO10-CBM10	31.6 ± 5.36	16.2 ± 4.41	28.5 ± 7.45
TbLPMO10-CBM10	17.21 ± 4.13	4.57 ± 3.19	14.4 ± 7.69
CfLPMO10-CBM3a	14.5 ± 3.11	6.94 ± 0.84	46.7 ± 17.16
TbLPMO10-CBM3a	ND*^a^*	ND*^a^*	ND*^a^*

*^a^* ND, no activity detected.

##### The CBMs Influence LPMO Activity

To explore the importance of the CBMs on the activity of the two LPMOs, truncated derivatives of *Tb*LPMO10 and *Cf*LPMO10 lacking these modules (defined as *Tb*LPMO10_CD_ and *Cf*LPMO10_CD_, respectively) were generated. The activities of the catalytic modules were generally markedly different from the corresponding wild type enzymes with respect to both the initial rate and the amount of limit product (total amount of gluconic acid generated when the reaction has gone to completion) (Fig. 1 and Table 1). For example, when compared with *Cf*LPMO10, *Cf*LPMO10_CD_ generated ∼4-fold less limit product, which was mirrored by a substantial reduction in the initial rate. Against Avicel and PASC, *Tb*LPMO10_CD_ displayed almost no detectable activity. The two exceptions to this trend were the activity of *Cf*LPMO10_CD_ and *Tb*LPMO10_CD_ against PASC and BMCC, respectively. Removal of the CBM from *Cf*LPMO10 caused a modest reduction in the initial rate (2-fold) and had very little impact on the quantity of limit products from the disordered cellulose. *Tb*LPMO10_CD_ displays the same initial rate as *Tb*LPMO10, but the truncated enzyme generates half the limit products from the BMCC. This may suggest that the active sites of *Cf*LPMO10_CD_ and *Tb*LPMO10_CD_ are optimized to bind highly exposed but insoluble cellulose structures, present in PASC, or the narrow crystalline surface presented by BMCC, respectively.

**FIGURE 4.**
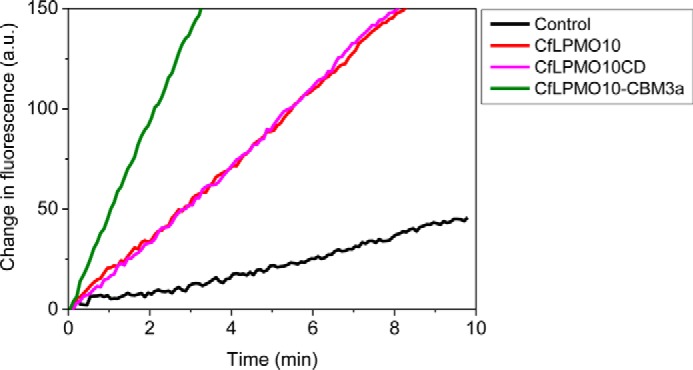
**Fluorometric assay for the generation of hydrogen peroxide by variants of *Cf*LPMO10.** *a.u.*, arbitrary units.

**FIGURE 5.**
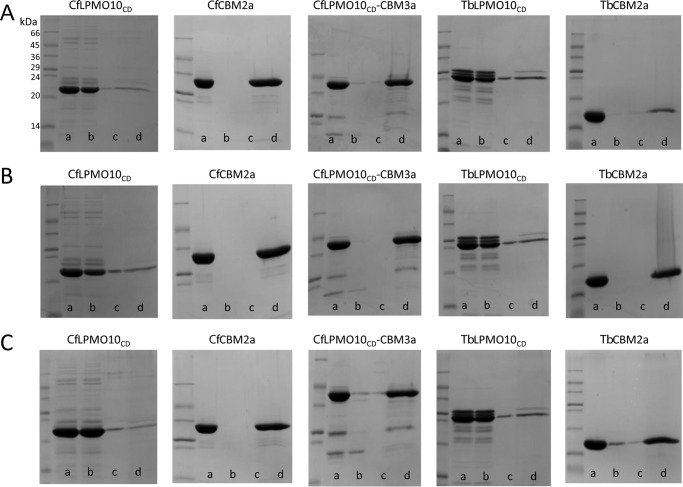
**Qualitative cellulose binding assays.** *A*, *B*, and *C*, binding to Avicel (5% (w/v)), PASC (1% (w/v)), and BMCC (0.35% (w/v)), respectively. *Lane a*, starting material; *lane b*, non-bound material in the supernatant after the cellulose had been pelleted; *lanes c* and *d*, wash (*lane c*) and material eluted from washed and pelleted cellulose by boiling in 10% SDS (*lane d*). Experiments were carried out on ice using 80 μg of proteins in 200 μl of 20 mm sodium phosphate buffer, pH 8.0. 10 mm EDTA was added to the samples containing *Cf*LPMO10_CD_ and *Tb*LPMO10_CD_ to prevent any catalytic activity.

It is possible that the reduced activity of *Tb*LPMO10_CD_ and *Cf*LPMO10_CD_ against the different forms of cellulose may reflect a loss of structural integrity of the enzyme. To test this hypothesis, a recently described assay (37) was employed to assess the production of H_2_O_2_ by the LPMOs. H_2_O_2_ coupled with horseradish peroxidase converts Amplex Red (10-acetyl-3,7-dihydroxyphenoxazine) to Resorufin, which was monitored using fluorescence. The data, presented in Fig. 4, show that the increase in fluorescence mediated by LPMOs with and without their native CBMs is similar. This indicates that the catalytic competence of the enzymes was not affected by the deletion of these non-catalytic modules. Indeed, given that CBMs and the catalytic modules of plant cell wall-degrading enzymes including LPMOs can be expressed as discrete entities and are generally separated by extended flexible linker sequences, it is generally accepted that these modules in full-length enzymes fold independent of each other (see Ref. 14 for review). These data indicate that the CBMs of *Tb*LPMO10 and *Cf*LPMO10 influence enzyme activity by contributing to substrate recognition. To explore this proposal further, the ligand specificity of the CBM2as derived from *Tb*LPMO10 and *Cf*LPMO10, *Tb*CBM2a and *Cf*CBM2a, respectively, was evaluated. The two CBMs bound to crystalline and acid-treated cellulose (Fig. 5), similar to other type A CBMs (41–43). The binding of *Tb*LPMO10_CD_ and *Cf*LPMO10_CD_ to PASC, Avicel, and BMCC was evaluated in the presence of EDTA and absence of ascorbate, to ensure that the enzymes were not catalytically competent. Surprisingly, *Tb*LPMO10_CD_ and *Cf*LPMO10_CD_ did not bind to cellulose (Fig. 5), supporting the hypothesis that the CBM2as play an important role in promoting prolonged enzyme-substrate binding. Forsberg *et al.* (44) also showed that the catalytic module of the cellulose-specific AA10 LPMO CelS2 failed to bind to cellulose, although it did bind to chitin. It was suggested that specificity in LPMOs is not conferred by distal subsites, but by the copper-containing active site and the geometry of substrate binding at the catalytic center. It should be noted, however, that the CBM2a in CelS2 appeared to display unusually weak binding to cellulose in the context of the full-length enzyme, and this may explain why its contribution to enzyme activity is modest (13).

**FIGURE 6.**
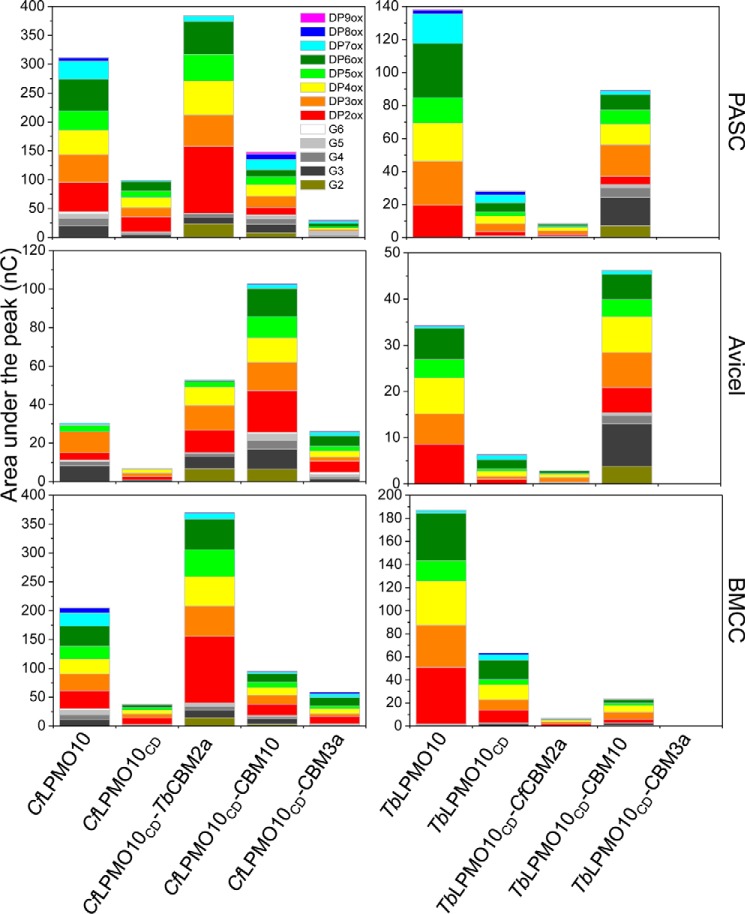
**Quantification of HPAEC analysis of limit products released from different forms of cellulose.** *G3*, *G4*, *G5*, and *G6* are cellotriose, cellotetraose, cellopentaose, and cellohexaose, respectively.

The reaction products generated by *Tb*LPMO10_CD_ and *Cf*LPMO10_CD_ from the three cellulose substrates were analyzed by HPAEC. The data, presented in supplemental Figs. S1 and S2 and Fig. 6, showed that the product profiles generated by *Tb*LPMO10 and *Tb*LPMO10_CD_ were similar. In contrast, *Cf*LPMO10_CD_ did not produce significant quantities of non-oxidized cello-oligosaccharides. This change in product profile is discussed in detail below.

##### The Activity of LPMO CBM Hybrids

**FIGURE 7.**
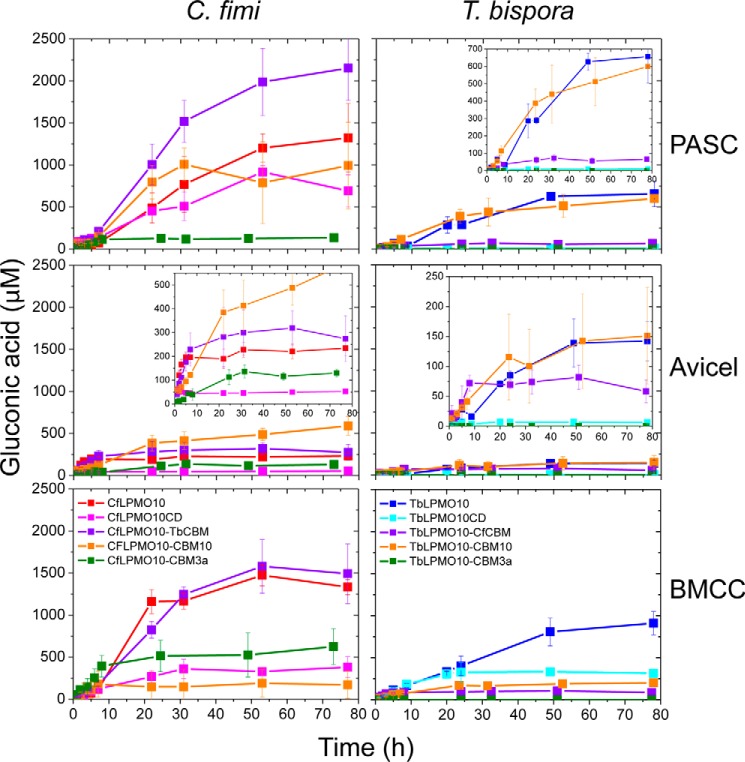
**Gluconic acid produced from different substrates.** The chromatographs for each time point are shown in supplemental Fig. S1 and supplemental Fig. S2. *Error bars* indicate means ± S.E.

The activity of glycoside hydrolases that attack cellulose and other components of the plant cell wall was also potentiated by CBMs (18, 19, 30, 41). The enhanced activity mediated by CBMs appears to simply reflect their affinity and binding capacity. To evaluate whether these principles also apply to LPMOs, the oxygenases were coupled to a variety of heterologous cellulose-specific CBMs, and the activity of the resultant enzymes was evaluated. The data, presented in supplemental Figs. S3–S6 and Fig. 7, show that the influence of CBMs on LPMO activity was enzyme- and substrate-specific. For example, replacing *Tb*CBM2a with *Cf*CBM2a in the *Thermobispora* LPMO resulted in a substantial reduction in activity against PASC and BMCC. In contrast, substitution of the *Cf*CBM2a with *Cj*CBM10 from *C. japonicus Cj*LPMO10B (34) enhanced the activity of *Cf*LPMO10 against Avicel, but greatly decreased the catalytic competence of both the *Cellulomonas* and *Thermobispora* oxygenases against BMCC. Significantly, swapping the endogenous CBM in *Tb*LPMO10 with *Cj*CBM10 greatly increased the quantity of non-oxidized oligosaccharides (discussed in detail below). It is possible that the differences in the activity profiles of the LPMO-CBM2a/10 fusions reflect the targeting of discrete regions of the three cellulosic substrates. To test this hypothesis, we evaluated whether combining LPMO-CBM fusions with different activity profiles increased the total amount of product generated. The data showed no synergistic or additive interactions with respect to the amount of limit products generated (data not shown). This suggests that the binding sites for the CBM2as and CBM10 are in close proximity and likely overlap. In contrast, synergistic interactions have been observed between actinomycete AA10 LPMOs (13) with respect to the rate of product release. As the quantity of limit products was not reported, however, it is uncertain whether these synergistic interactions led to an increase in the limit products.

When the CBM2as were exchanged for CBM3a from *C. thermocellum* CipA, there was a substantial reduction in the activity of both LPMOs. Indeed, no significant activity was detected for *Tb*LPMO10_CD_-CBM3a against all three forms of cellulose and for *Cf*LPMO10_CD_-CBM3a against PASC. The CBM3a-mediated reduction in activity did not reflect an interaction between the hydrophobic surface of the LPMOs and the ligand binding site of the CBM as *Cf*LPMO10_CD_-CBM3a, but not *Cf*LPMO10_CD_, bound to the three forms of cellulose (Fig. 5). Furthermore, the active site of the enzyme in the CBM3a-LPMO fusion was functional, as the chimeric enzyme is still able to reduce O_2_ to H_2_O_2_.

To explore whether there is a relationship between the binding properties of the CBMs and their influence on oxygenase activity, the affinity and binding capacity of these protein modules were explored. The data, presented in Table 2, showed that there was an inverse relationship between affinity and binding capacity and that the two CBMs from thermophilic organisms, CBM3a and *Tb*CBM2a, bound tighter than the proteins from mesophilic bacteria. In short, the binding profiles provide no obvious insight into the influence of the CBMs on LPMO activity.

**TABLE 2:** **Binding of CBMs to cellulose** Measurements were performed in 50 mm sodium phosphate buffer, pH 6.0.

	Saturation	*K_d_*
	μ*mol/g of cellulose*	μ*m*
**PASC**		
*Tb*CBM2	14.73 ± 3.36	1.61 ± 0.72
*Cf*CBM2	29.58 ± 3.24	5.83 ± 1.16
*Ct*CBM3a	12.32 ± 1.48	3.21 ± 1.17
*Cj*CBM10	47.14 ± 2.67	17.34 ± 3.54

**Avicel**		
*Tb*CBM2	4.44 ± 1.16	3.16 ± 2.33
*Cf*CBM2	9.94 ± 3.21	7.29 ± 3.63
*Ct*CBM3a	2.25 ± 1.06	3.33 ± 1.77
*Cj*CBM10	12.93 ± 0.93	7.49 ± 1.47

**BMCC**		
*Tb*CBM2	17.03 ± 1.73	1.81 ± 0.82
*Cf*CBM2	25.29 ± 5.18	3.47 ± 1.40
*Ct*CBM3a	13.76 ± 0.20	2.75 ± 0.26
*Cj*CBM10	45.72 ± 6.07	11.41 ± 1.80

##### Influence of CBMs on Synergy between LPMO10s and Cellulases

**FIGURE 8.**
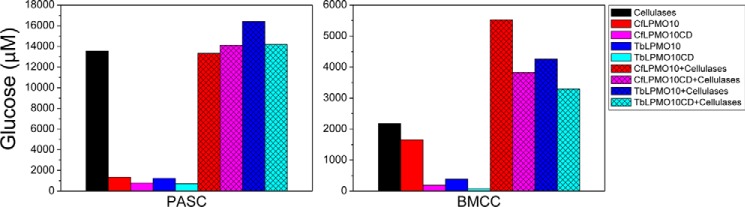
**Synergy between LPMOs and cellulases *Cj*Cel6A and *Cj*Cel5B.** The data shown are for 100-h incubations of PASC and BMCC with the enzymes indicated. LPMOs and cellulases were used at 1 and 0.5 μm, respectively.

Previous studies showed that a number of LPMOs potentiate the activity of cellulases and chitinases against their respective insoluble substrates (5, 7, 46). The role of CBMs in the synergistic interactions between LPMOs and glycanases is poorly understood. To address this issue, the capacity of *Cf*LPMO10 fused to different CBMs to act in synergy with a cellobiohydrolase (*Cj*Cel6A) and an endoglucanase (*Cj*Cel5B) (34) was explored. The data, presented in Fig. 8, showed that there was a significant increase in the amount of limit glucose released from BMCC when the cellulases and the unmodified LPMOs were combined, when compared with when these enzymes were incubated individually with this crystalline form of cellulose. The synergy between the glycoside hydrolases and oxygenases was not apparent when PASC was used as the substrate. Synergistic interactions between the cellulases and truncated forms of the LPMOs lacking their CBMs were evident, when evaluated against BMCC, although the total amount of glucose generated was reduced. These data demonstrate that the LPMOs exhibit synergistic interactions with cellulases, but the functional interactions between these enzymes were not dependent on the presence of CBMs in the oxygenases.

## Discussion

The data presented here show that the CBMs of two LPMO10s contribute to the activity of the enzymes against different forms of cellulose. The effect of the CBMs varied dependent on the cellulosic substrate and the enzyme. Indeed, the observation that the catalytic domains of both LPMOs displayed no prolonged binding to the three forms of insoluble cellulose used here suggests that retention of the LPMOs on the surface of the substrate was mediated by the appended CBMs. It should be emphasized that the presence of CBMs is not a universal feature of LPMOs that attack crystalline polysaccharides (47), and thus these modules are not integral to the function of all oxygenases that target recalcitrant substrates. This implies that there is significant variation in the capacity of the catalytic modules of LPMOs to bind their respective substrates. The first LPMO identified, CBP21, lacked a CBM and bound tightly to its substrate chitin (32). Indeed, this enzyme was initially thought to be a CBM that potentiated the activity of chitinases through a non-catalytic mechanism (46). Similarly, an AA9 LPMO from *Neurospora crassa* (GenBank^TM^ accession number NCU03328; *Nc*LPMO9_03328_) also lacked a CBM but attacked crystalline cellulose (48). Significantly, CBM1-containing AA9 LPMOs from *Podospora anserina* generated 3–4-fold more soluble products from cellulose than other LPMO9s derived from the fungus that contained no CBM (47). This suggests that the substrate binding capacities of the catalytic domains of at least some LPMOs lacking a *natural* CBM do not fully compensate for the absence of these non-catalytic cellulose targeting modules.

The CBM fusion experiments again showed that the effects of these modules were substrate- and enzyme-specific. The activity of *Tb*LPMO10 could not be improved by introducing a different CBM, suggesting that its *natural* non-catalytic module, *Tb*CBM2a, is optimal for this enzyme. In contrast, the observation that *Cj*CBM10 and *Tb*CBM2a improved the activity of *Cf*LPMO10 against crystalline cellulose suggests that the activity of at least some lytic monooxygenases can be enhanced by CBM swapping.

An intriguing feature of the CBM truncation and swapping experiments was the change in the ratio of oxidized and non-oxidized products. Thus, removal of the endogenous CBM from *Cf*LPMO10 caused a reduction in the ratio of oxidized to non-oxidized oligosaccharides. Replacing the endogenous CBM2a from *Tb*LPMO10 with *Cj*CBM10 resulted in a substantial increase in the ratio of oxidized to non-oxidized products. The origin of the non-oxidized products is unclear. It is possible that these oligosaccharides are generated by C1 lytic oxidations near the reducing end of cellulose chains. Alternatively, at least for wild type *Cf*LPMO10, non-oxidized products could have occurred when the enzyme mediated C1 oxidative cleavage and a downstream (toward the reducing end) C4 lytic oxidation. Based on this logic, removal of the CBM greatly reduced the capacity of the enzyme to cleave C4–H bond when compared with C1–H. In this scenario, the positioning of O–C4 linkage in the active site would be dependent on the binding of *Cf*CBM2a to the substrate in a specific register with respect to the catalytic module. Although not quantitative, the respective mass spectrometry and HPAEC signals for the C4+1- and C4-oxidized species, generated by wild type *Cf*LPMO10, were very small when compared with the C1-oxidized oligosaccharides. If the proposed imbalance between C1- and C4-oxidized products is correct, then it is unlikely that the significant quantities of non-oxidized species were generated from lytic oxidation at C4 and C1, as this would result in stoichiometric amounts of uncharged and doubly oxidized species. Thus, we believe that the non-oxidized species are derived from oligosaccharides released from the reducing end of the cellulose chains. With respect to *Tb*LPMO10_CD_-CBM10, it is interesting to note that the wild type *Thermobispora* enzyme generates exclusively C1-oxidized products, whereas *Cj*LPMO10B produces modest amounts of non-oxidized species (34). It would appear, therefore, that the properties of the hybrid LPMO are quite different from the progenitor enzymes. It is unclear how *Cj*CBM10 modulates the mode of action of *Tb*LPMO10, but we propose that the binding module directs the enzyme toward the reducing ends of cellulose chains where cleavage is mediated by C1 oxidation.

The negative impact of CBM3a on the activity of the LPMOs is surprising, as several studies have shown that this module potentiates the activity of cellulases and other glycanases that attack the plant cell wall (18, 19, 49). It is possible that the CBM3a targets the LPMOs to regions of the cellulose substrates that are not accessible to the active site of these enzymes. Carrard *et al.* (49) showed that CBM3a could direct cellulases to regions of crystalline substrates that were not accessible to other type A CBMs. The substrate binding cleft of cellulases is optimized to bind isolated cellulose chains, whereas LPMOs act on microfibrils. Thus, the CBM3a may target LPMOs to regions of cellulose that are accessible to cellulases but not to the lytic oxygenases used here, resulting in the formation of non-productive complexes between substrate and the lytic oxygenase. The ligand binding site of type A CBMs comprises a planar hydrophobic surface (see Refs. 14 and 15 for review). The differing effects of type A CBMs on the activity of the LPMOs suggest that these modules can target subtle differences in the planar surfaces presented by cellulose microfibrils. This is consistent with variation in binding sites occupied by type A cellulose-specific CBMs on purified forms of the polysaccharide (50) and in plant cell walls (45). Conversely, the effect of several CBMs was LPMO-specific, indicating fundamental differences in substrate binding by the catalytic domains of these enzymes. In this regard, Beeson *et al.* (28) have proposed that the spatial position of the aromatic residues in the planar surface of AA9 LPMOs indicates that some enzymes can bind along a single chain of cellulose on the microfibrils, whereas others adopt a perpendicular orientation across the trajectory of the polymer. In AA10 enzymes, the apparent binding surface is dominated by hydrophilic residues with only a single conserved aromatic amino acid, which is also evident in AA9 enzymes. The mechanism by which the bacterial LPMOs interact with substrate in the absence of CBMs remains opaque.

This study shows that cellulose binding CBMs can have a significant influence on LPMO activity, and that their effect varies dependent on the source of these modules, the enzyme used, and the substrate evaluated. These data show that the mechanisms by which CBMs enhance LPMO activity are more complex than simply promoting enzyme substrate proximity, as occurs in glycoside hydrolases (cellulases). The interplay between CBMs and LPMOs is an important area to explore further as we design efficient bespoke hybrid enzymes.

## Author Contributions

L. I. C. analyzed the biochemistry of LPMOs. A. L. characterized CBMs. G. J. D. and P. H. W. provided intellectual insight into enzyme function. H. J. G. supervised the work and contributed to writing the manuscript.

## Supplementary Material

Supplemental Data

## Footnotes

3 The abbreviations used are:

LPMO: lytic polysaccharide monooxygenase
CBM: non-catalytic carbohydrate binding module
PASC: phosphoric swollen-acid cellulose
BMCC: bacterial crystalline cellulose
AA: auxiliary activity
HPAEC: high performance anion exchange chromatography
DP: degree of polymerization.
