# Supplementary material for: The Contribution of Non-catalytic Carbohydrate Binding Modules to the Activity of Lytic Polysaccharide Monooxygenases
Source: J Biol Chem. 2016 Jan 22;291(14):7439–49. doi: 10.1074/jbc.M115.702365 (PMC4817175; doi:10.1074/jbc.M115.702365)
Supplement: Supplemental Data [file supp_291_14_7439__index.html]

The contribution of non-catalytic carbohydrate binding modules to the activity lytic polysaccharide monooxygenases — The Contribution of Non-catalytic Carbohydrate Binding Modules to the Activity of Lytic Polysaccharide Monooxygenases — CBMs and LPMOs — Supplemental Data 

# The Contribution of Non-catalytic Carbohydrate Binding Modules to the Activity of Lytic Polysaccharide Monooxygenases

## Supplemental Data

- All supplemental figures (.pdf, 1.3 MB) - All supplemental figures
