# Supplementary material for: The Contribution of Non-catalytic Carbohydrate Binding Modules to the Activity of Lytic Polysaccharide Monooxygenases
Source: J Biol Chem. 2016 Jan 22;291(14):7439–49. doi: 10.1074/jbc.M115.702365 (PMC4817175; doi:10.1074/jbc.M115.702365)
Supplement: Supplemental Data [file 10.1074_M115.702365_jbc.M115.702365-1.pdf]

Supplementary figures for

The contribution of non-catalytic carbohydrate binding modules to the activity lytic polysaccharide monooxygenases

Lucy I Crouch[1¶], Aurore Labourel[1¶], Paul H. Walton[2], Gideon J. Davies[2] and Harry J. Gilbert[1*]

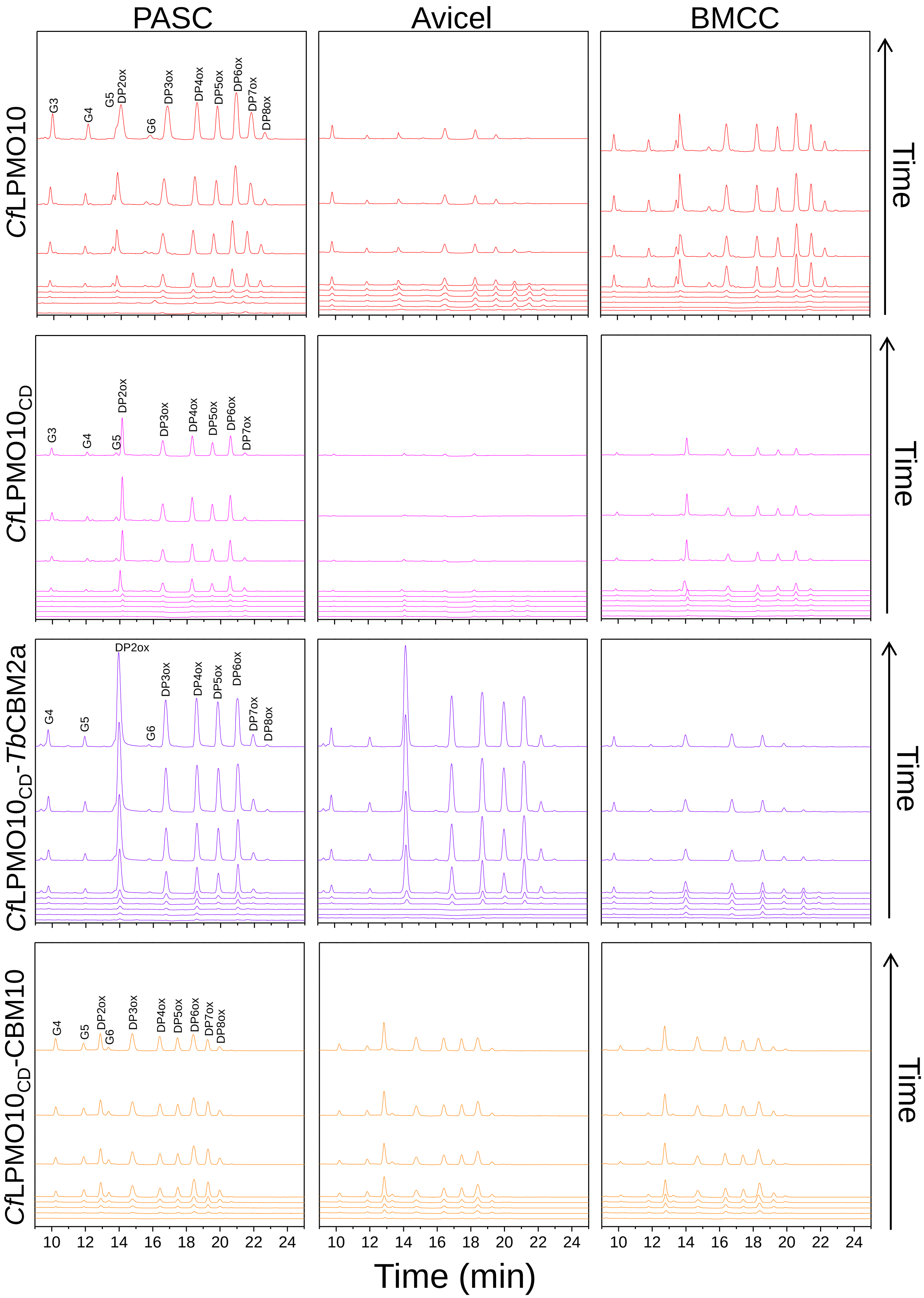

Fig. S1. HPEAC analysis of the soluble products produced from three cellulose substrates by the different *C. fimi* LPMOs. A chromatogram is provided for each time point corresponding to the time points indicated to in Figure 7, with the earliest at the bottom and the latest at the top of each panel.

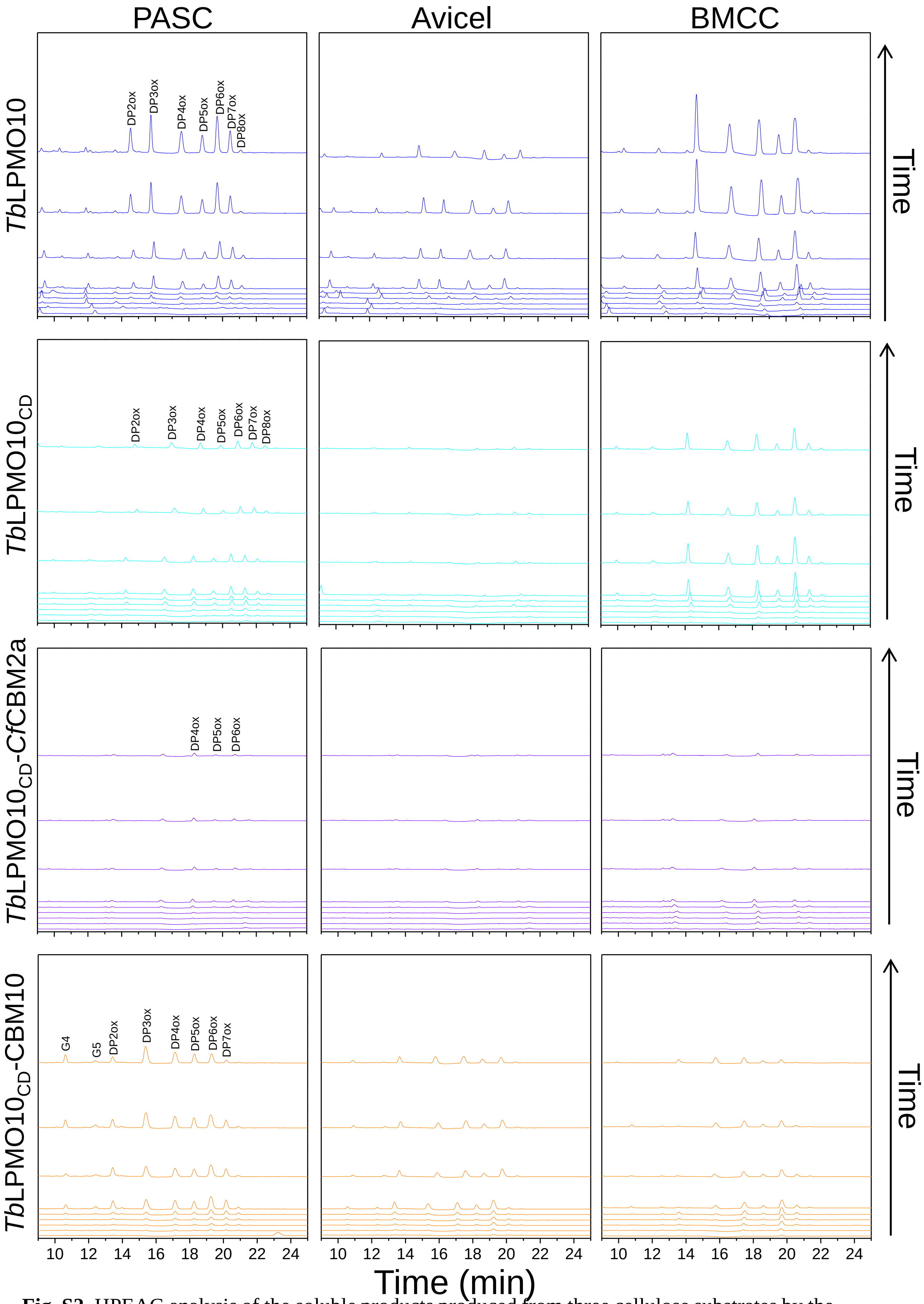

Fig. S2. HPEAC analysis of the soluble products produced from three cellulose substrates by the different *T. bispora* constructs. A chromatogram is provided for each time point corresponding to the time points indicated to in Figure 7, with the earliest at the bottom and the latest at the top of each panel.

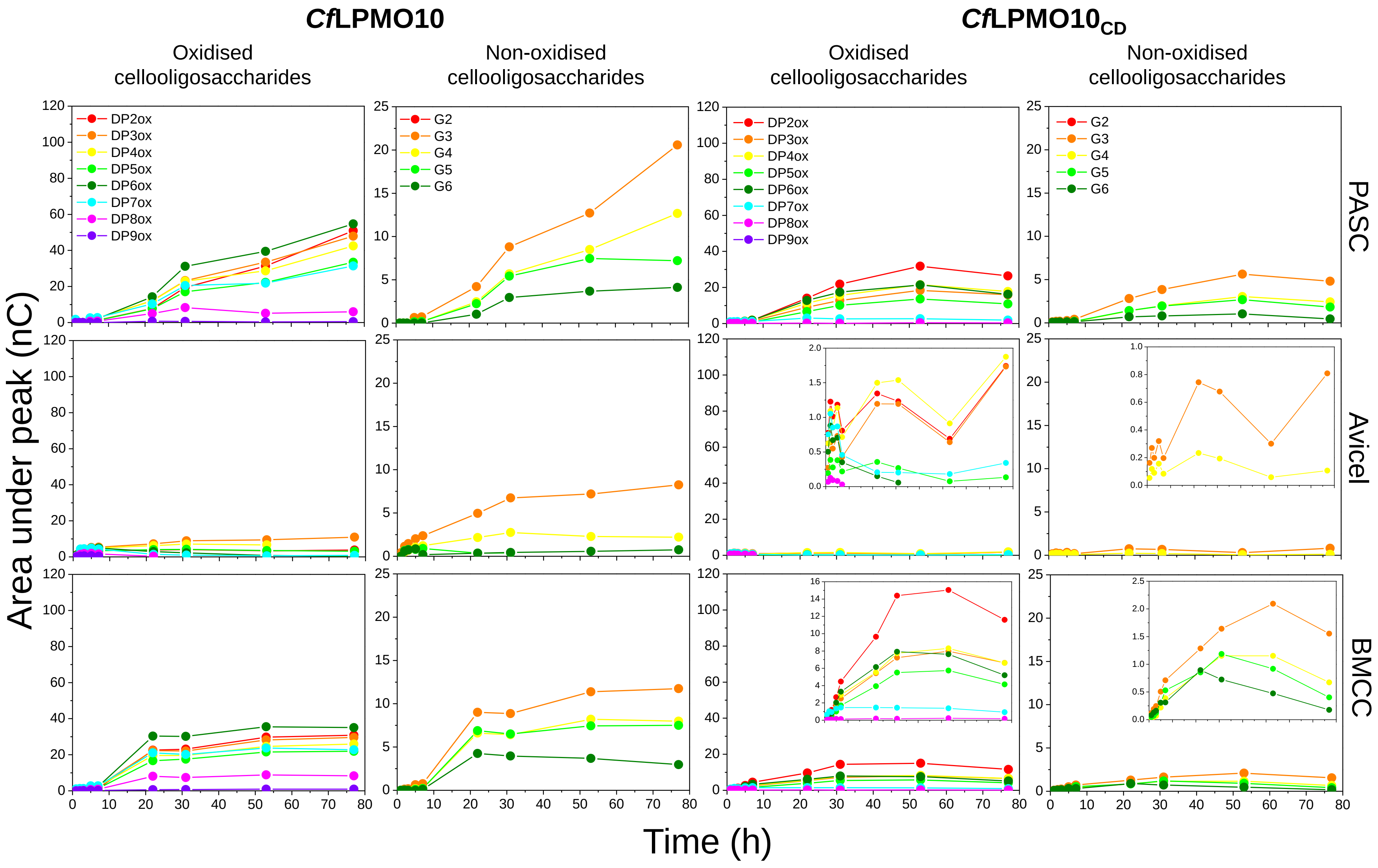

Fig. S3. Quantification of the soluble oxidised and non-oxidised products released from different substrates by *Cf*LPMO10 and *Cf*LPMO10$_{CD}$.

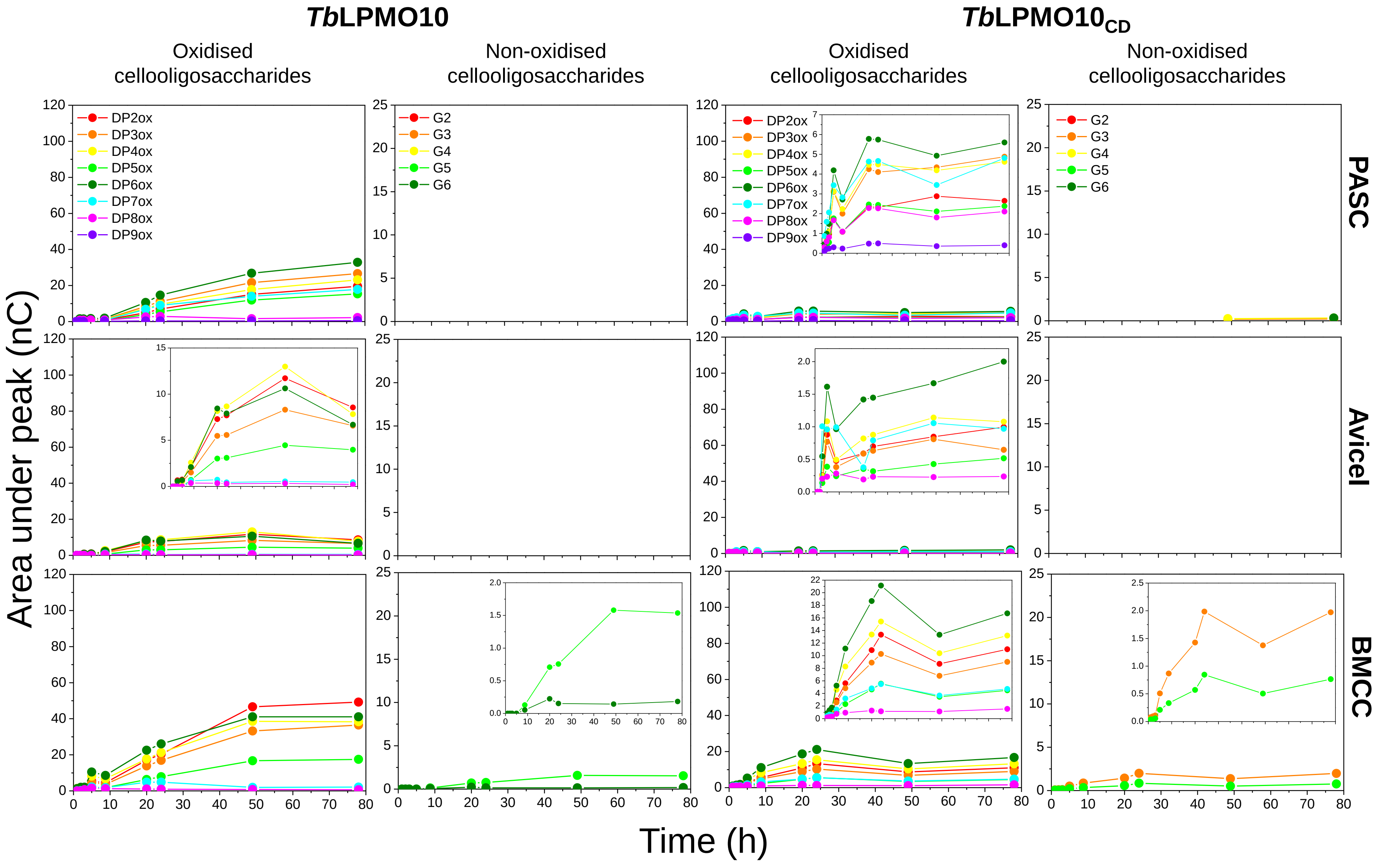

Fig. S4. Quantification of the soluble oxidised and non-oxidised products released from different substrates by *Tb*LPMO10 and *Tb*LPMO10$_{CD}$.

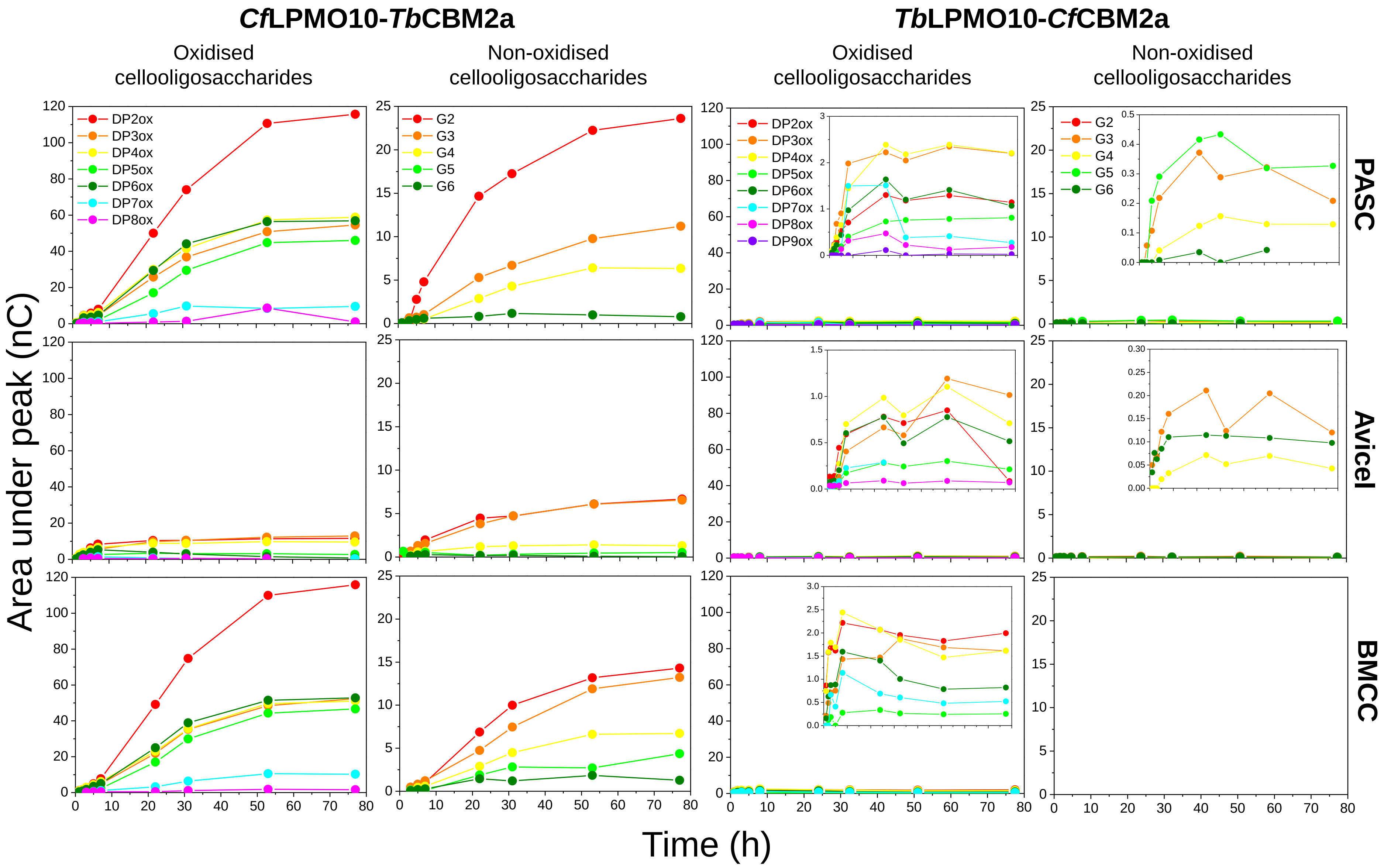

Fig. S5. Quantification of the soluble oxidised and non-oxidised products released from different substrates by *Cf*LPMO10-*Tb*CBM2a and *Tb*LPMO10-*Cf*CBM2a.

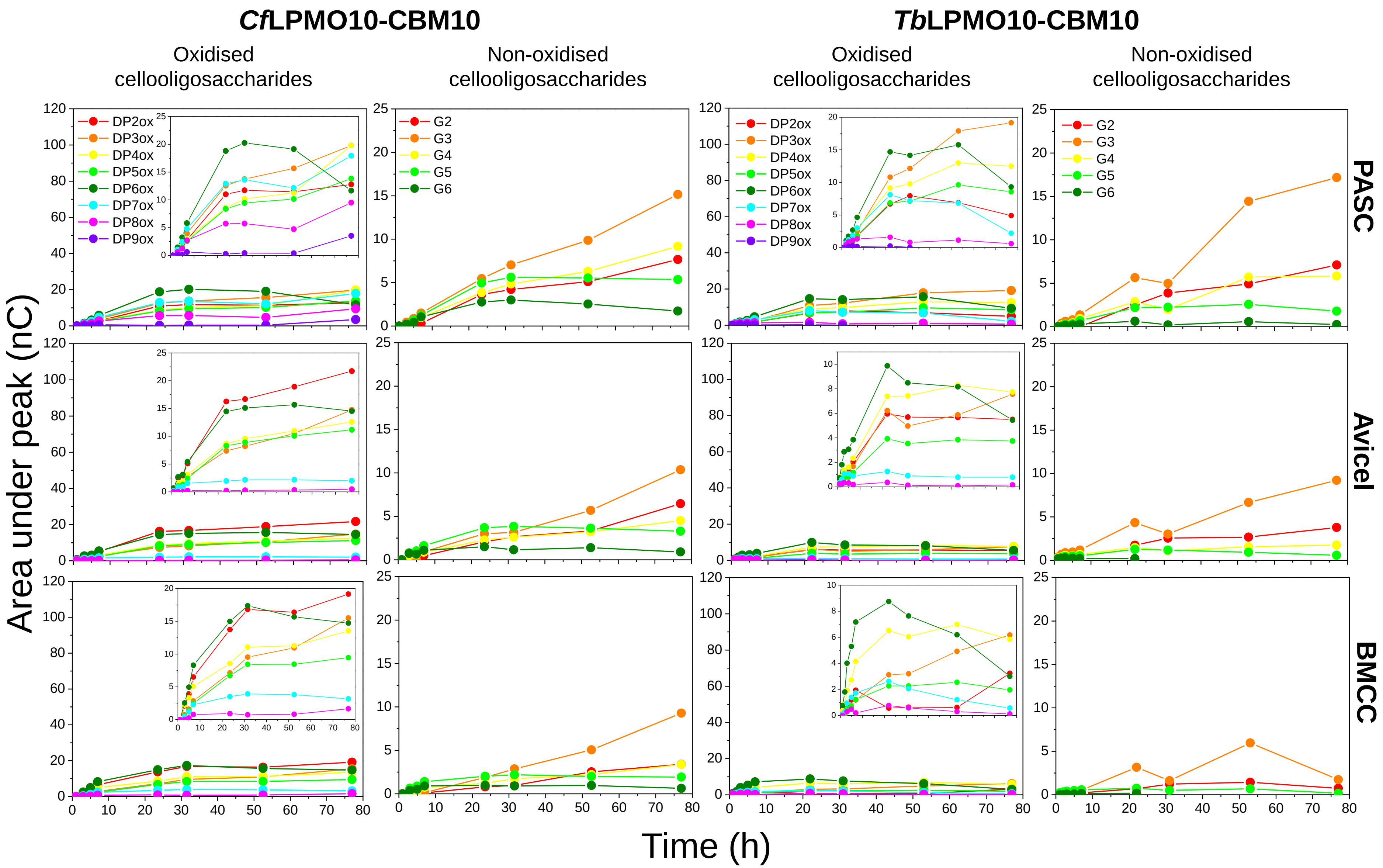

Fig. S6. Quantification of the soluble oxidised and non-oxidised products released from different substrates by *Cf*LPMO10-CBM10 and *Tb*LPMO10-CBM10.
